# Supplementary material for: Dexamethasone and lactoferrin induced PMN-MDSCs relieved inflammatory adverse events of anti-cancer therapy without tumor promotion
Source: Commun Biol. 2021 Feb 26;4:252. doi: 10.1038/s42003-021-01769-z (PMC7910613; doi:10.1038/s42003-021-01769-z)
Supplement: Supplementary file 3 — Description of Additional Supplementary Files [file 42003_2021_1769_MOESM3_ESM.pdf]

Description of Additional Supplementary Files

File name: Supplementary Data 1

Description: The source data underlying all the statistical analysis in the main figures and supplementary figures. They were displayed as tables in Excel form with the names according to their related figures.
