# Supplementary material for: Dexamethasone and lactoferrin induced PMN-MDSCs relieved inflammatory adverse events of anti-cancer therapy without tumor promotion
Source: Commun Biol. 2021 Feb 26;4:252. doi: 10.1038/s42003-021-01769-z (PMC7910613; doi:10.1038/s42003-021-01769-z)
Supplement: Supplementary file 5 — Reporting Summary [file 42003_2021_1769_MOESM5_ESM.pdf]

nature research

Corresponding author(s): Xing Li; Yan-Fang Xing; Xiang-Yuan Wu

Last updated by author(s): Dec 16, 2020

Reporting Summary

Nature Research wishes to improve the reproducibility of the work that we publish. This form provides structure for consistency and transparency in reporting. For further information on Nature Research policies, see our Editorial Policies and the Editorial Policy Checklist.

Statistics

For all statistical analyses, confirm that the following items are present in the figure legend, table legend, main text, or Methods section.

n/a	Confirmed	
☐	☒	The exact sample size (n) for each experimental group/condition, given as a discrete number and unit of measurement
☐	☒	A statement on whether measurements were taken from distinct samples or whether the same sample was measured repeatedly
☐	☒	The statistical test(s) used AND whether they are one- or two-sided<br>*Only common tests should be described solely by name; describe more complex techniques in the Methods section.*
☐	☒	A description of all covariates tested
☒	☐	A description of any assumptions or corrections, such as tests of normality and adjustment for multiple comparisons
☐	☒	A full description of the statistical parameters including central tendency (e.g. means) or other basic estimates (e.g. regression coefficient) AND variation (e.g. standard deviation) or associated estimates of uncertainty (e.g. confidence intervals)
☐	☒	For null hypothesis testing, the test statistic (e.g. F, t, r) with confidence intervals, effect sizes, degrees of freedom and P value noted<br>*Give P values as exact values whenever suitable.*
☒	☐	For Bayesian analysis, information on the choice of priors and Markov chain Monte Carlo settings
☒	☐	For hierarchical and complex designs, identification of the appropriate level for tests and full reporting of outcomes
☒	☐	Estimates of effect sizes (e.g. Cohen's d, Pearson's r), indicating how they were calculated

Our web collection on statistics for biologists contains articles on many of the points above.

Software and code

Policy information about availability of computer code

Data collection	no software used
Data analysis	GraphPad Prism version 7.0 and SPSS Statistics 20.0

For manuscripts utilizing custom algorithms or software that are central to the research but not yet described in published literature, software must be made available to editors and reviewers. We strongly encourage code deposition in a community repository (e.g. GitHub). See the Nature Research guidelines for submitting code & software for further information.

Data

Policy information about availability of data

All manuscripts must include a data availability statement. This statement should provide the following information, where applicable:

- Accession codes, unique identifiers, or web links for publicly available datasets
- A list of figures that have associated raw data
- A description of any restrictions on data availability

The authors declare that [the/all other] data supporting the findings of this study are available within the paper and its supplementary information files.

Field-specific reporting

Life sciences study design

All studies must disclose on these points even when the disclosure is negative.

Sample size	n=6 in in vivo study. n=4-6 in in vitro study according to most similar studies.
Data exclusions	n/a
Replication	the experiments were repeated three times and the results were repeatable.
Randomization	the samples are randomized using the method of random number table
Blinding	the investigators were blinded to the experiment groups

Reporting for specific materials, systems and methods

We require information from authors about some types of materials, experimental systems and methods used in many studies. Here, indicate whether each material, system or method listed is relevant to your study. If you are not sure if a list item applies to your research, read the appropriate section before selecting a response.

Materials & experimental systems

n/a	Involved in the study	
☐	☒	Antibodies
☒	☐	Eukaryotic cell lines
☒	☐	Palaeontology and archaeology
☐	☒	Animals and other organisms
☐	☒	Human research participants
☒	☐	Clinical data
☒	☐	Dual use research of concern

Methods

n/a	Involved in the study	
☒	☐	ChIP-seq
☐	☒	Flow cytometry
☒	☐	MRI-based neuroimaging

Antibodies

Antibodies used	antibodies used for flow cytometer and western blot analysis were listed in Table S2
Validation	validation of the antibodies were on the manufacturer home page and can be found with the catalog number

Animals and other organisms

Policy information about studies involving animals; ARRIVE guidelines recommended for reporting animal research

Laboratory animals	C57BL/6 and BALB/C mice
Wild animals	n/a
Field-collected samples	n/a
Ethics oversight	The animal study was approved by the Institutional Animal Care and Use Committee of the Sun Yat-Sen University.

Note that full information on the approval of the study protocol must also be provided in the manuscript.

Human research participants

Policy information about studies involving human research participants

Population characteristics	Blood samples were collected from eighteen adult healthy donors and six late stage HCC patients between February, 2019 and July, 2019 at the Third Affiliated Hospital of Sun Yat-Sen University, Guangzhou, China.
Recruitment	there were included in clinical practice
Ethics oversight	This study was approved by the Clinical Ethics Review Board of the Third Affiliated Hospital of Sun Yat-Sen University. A written informed consent was obtained from all the patients at the time of admission.

Note that full information on the approval of the study protocol must also be provided in the manuscript.

Flow Cytometry

Plots

Confirm that:

- [x] The axis labels state the marker and fluorochrome used (e.g. CD4-FITC).
- [x] The axis scales are clearly visible. Include numbers along axes only for bottom left plot of group (a 'group' is an analysis of identical markers).
- [x] All plots are contour plots with outliers or pseudocolor plots.
- [x] A numerical value for number of cells or percentage (with statistics) is provided.

Methodology

Sample preparation	PBMCs of HCC patients and mononuclear cells from mice samples were isolated by Ficoll centrifugation.
Instrument	FACSAria II flow cytometer (BD Bioscience)
Software	FlowJo V10.0.7 (FlowJo, OR, USA).
Cell population abundance	> 30000 cells are needed for each analysis
Gating strategy	it was added in supplementary figure 1

- [x] Tick this box to confirm that a figure exemplifying the gating strategy is provided in the Supplementary Information.
