# Supplementary material for: Dexamethasone and lactoferrin induced PMN-MDSCs relieved inflammatory adverse events of anti-cancer therapy without tumor promotion
Source: Commun Biol. 2021 Feb 26;4:252. doi: 10.1038/s42003-021-01769-z (PMC7910613; doi:10.1038/s42003-021-01769-z)
Supplement: Supplementary file 1 — Supplementary Information [file 42003_2021_1769_MOESM1_ESM.pdf]

Reviewers' comments:

Reviewer #1 (Remarks to the Author):

In this manuscript "Dexamethasone and lactoferrin induced PMN-MDSCs relieved inflammatory adverse events of anti-cancer therapy without tumor promotion", the authors discuss the role of dexamethasone and lactoferrin in inducing PMN-MDSC production. In their studies, the authors found that DXM/LF PMN-MDSCs were distinct from tumor PMN-MDSCs, and these cells relieved cisplatin-induced acute kidney failure, bleomycin-induced interstitial pneumonia, and allergic pneumonitis effectively without promoting tumor development.
Major concerns:
1 The author should provide the gate strategy of flow cytometry.
2 In Fig1D and Fig 2D, the image is not clear, and the ruler cannot be distinguished.
3 In line 86, PMN-MDSCs from hepatocellular carcinoma (HCC) patients, the author should show how to obtain these PMN-MDSCs.
4 In line 99, spleen PMN-MDSCs from DXM/LF group? Are these cells immunosuppressive function? How to determine whether it is a PMN-MDSC?
5 In line 122, cited reference.
6 Is DXM regulating Lrp1expression on myeloid cell or PMN-MDSC?

Reviewer #2 (Remarks to the Author):

This manuscript explored the role of DXM/LF-induced PMN-MDSCs in relieving inflammatory conditions using mouse models of cisplatin-induced acute kidney failure, bleomycin-induced interstitial pneumonia, and allergic pneumonitis with minimal effect on tumor growth. Although this study holds some levels of interests, it is not developed into scientifically sound contribution in its current form. The paper can be improved by addressing the specific comments below.

Specific comments:
1. Although the authors provided the clear evidence that adoptive transfer of DXM/LF-induced PMN-MDSCs reduces inflammatory conditions in mouse models of cisplatin-induced acute kidney failure, bleomycin-induced interstitial pneumonia, and allergic pneumonitis, the effect of DXM/LF-induced PMN-MDSCs on tumor development still remains vague especially given the artificial nature of the transplantable tumor model of B16 used. Furthermore, it has been established that PMN-MDSCs promote tumor development in each fundamental steps of cancer progression from primary tumor to metastatic disease. The role of DXM/LF-induced PMN-MDSCs in regulating tumor metastasis warrants at least investigation.
2. In addition, the authors found that compared to control and tumor PMN-MDSCs, DXM/LF-induced PMN-MDSCs produced significantly higher levels of PGE2 that has been documented well in inhibiting antitumor immunity. Thus, the effect of DXM/LF-induced PMN-MDSCs on both systemic and local antitumor immune responses needs to be examined.
3. The panel G in Fig. 7 is missing.

To Reviewer 1

1 The author should provide the gate strategy of flow cytometry.

Response: Thank you for this professional suggestion. We added the gate strategy of flow cytometry as supplementary figure.

2 In Fig1D and Fig 2D, the image is not clear, and the ruler cannot be distinguished.

Response: Thank you for this kind reminding. We increased the DPI of the figure from 150 to 600.

3 In line 86, PMN-MDSCs from hepatocellular carcinoma (HCC) patients, the author should show how to obtain these PMN-MDSCs.

Response: This is an excellence advice. We added this information in the results and methods section as follows:

Results

DXM and LF-induced human PMN-MDSC in vitro from human peripheral blood mononuclear cells (PBMCs)

Next, PBS- or DXM- and LF-induced PMN-MDSCs, and PMN-MDSCs sorted from PBMC of hepatocellular carcinoma (HCC) patients were collected to test their immuno-suppressive function. $CD3^+$ T cells from PBMCs were labeled with CFSE (2 μM), stimulated with anti-CD3–coated (5 μg/mL) plates and soluble anti-CD28 antibody (1 μg/mL), and cultured alone or with PMN-MDSCs at 2:1 ratio for 3 d.

4 In line 99, spleen PMN-MDSCs from DXM/LF group? Are these cells immunosuppressive function? How to determine whether it is a PMN-MDSC?

Response: We are sorry for ignorance of presenting this data. These cells are PMN-MDSC with immune suppressive function. The data were added in supplementary figures. We revised the manuscript as follows:

Results

DXM- and LF-induced PMN-MDSCs are distinct from tumor PMN-MDSCs

DXM- and LF-induced PMN-MDSCs in vivo presented suppressive function to T cell proliferation, which was slightly weaker than tumor PMN-MDSC (Fig. S2).

5 In line 122, cited reference.

Response: We added the reference as you demanded.

6 Is DXM regulating Lrp1expression on myeloid cell or PMN-MDSC?

Response: This is an excellent question. DXM and LF were used to induce PMN-MDSC from myeloid cells of the bone marrow of mice or the PBMC of human. Before these myeloid cells achieve immune suppressive function, they are defined as myeloid cell. Thus, we use "DXM upregulated myeloid cell response to LF" in the paper.

To reviewer 2

1. Although the authors provided the clear evidence that adoptive transfer of DXM/LF-induced PMN-MDSCs reduces inflammatory conditions in mouse models of cisplatin-induced acute kidney failure, bleomycin-induced interstitial pneumonia, and allergic pneumonitis, **the effect of DXM/LF-induced PMN-MDSCs on tumor development still remains vague especially given the artificial nature of the transplantable tumor model of B16 used. Furthermore, it has been established that PMN-MDSCs promote tumor development in each fundamental steps of cancer progression from primary tumor to metastatic disease. The role of DXM/LF-induced PMN-MDSCs in regulating tumor metastasis warrants at least investigation.**

Response: Thank you very much for this professional suggestion. Orthotopic mouse model of spontaneous breast cancer metastasis was utilized to evaluated the tumor promotion capacity of DXM/LF PMN-MDSCs. DXM/LF PMN-MDSCs, Con PMN-MDSCs, or tumor PMN-MDSCs were transferred of into 4T1 tumor bearing mice, when the subcutaneous tumors were between 0.9 – 1.1 cm in diameter three times every other day. PBS was used as vehicle control. Mice were tested 1 week after transfer. DXM/LF PMN-MDSCs did not promote tumor growth and metastasis. We revised our paper as follows:

Results

DXM/LF PMN-MDSCs presented improved survival capability, less tumor homing tendency and negative tumor promotive effects compared to tumor PMN-MDSCs

Orthotopic mouse model of spontaneous breast cancer metastasis was utilized to evaluated the tumor promotion capacity of DXM/LF PMN-MDSCs. DXM/LF PMN-MDSCs, Con PMN-MDSCs, or tumor PMN-MDSCs were transferred of into

4T1 tumor bearing mice, when the subcutaneous tumors were between 0.9 – 1.1 cm in diameter three times every other day. PBS was used as vehicle control. Mice were tested 1 week after transfer. transfer of tumor PMN-MDSCs and Con PMN-MDSCs promoted the growth of tumor significantly instead of DXM/LF PMN-MDSCs (Fig. 4F). Lung nodule area per mouse were highest in tumor PMN-MDSC group. Meanwhile, DXM/LF PMN-MDSCs group and Con PMN-MDSCs group did not promote lung metastasis (Fig. 4G and H). DXM/LF PMN-MDSCs did not promote tumor progression.

Discussion

Besides, DXM/LF PMN-MDSCs presented less tumor tissue homing, weaker immuno-suppression, and negative influence to antitumor immune response in tumor tissues. As a result, transfer of DXM/LF PMN-MDSCs, instead of Con PMN-MDSC, did not promote tumor metastasis and growth. Thus, DXM/LF PMN-MDSCs were different from the tumor PMN-MDSCs and presented reduced tendency to promote tumor progression. However, DXM/LF PMN-MDSCs transfer in this study was no more than 3 times or 5 days. The safety of long term usage of DXM/LF PMN-MDSCs transfer and its therapeutic regimen on human was still uncertain.

2. In addition, the authors found that compared to control and tumor PMN-MDSCs, DXM/LF-induced PMN-MDSCs produced significantly higher levels of PGE2 that has been documented well in inhibiting antitumor immunity. Thus, the effect of DXM/LF-induced PMN-MDSCs on both systemic and local antitumor immune responses needs to be examined.

Response: Thanks very much for this professional advice. To analyze the effect of DXM/LF-induced PMN-MDSCs on both systemic and local antitumor immune responses, IFN-γ^+ CD8 T cells in PBMC and tumor tissue were tested by flow cytometers 24 hour after transfer of DXM/LF PMN-MDSCs, Con PMN-MDSCs, or tumor PMN-MDSCs into B16 tumor bearing mice, when the tumors were between 0.9 – 1.1 cm in diameter. PBS was used as vehicle control. In PBMC, compared with PBS group, the IFN-γ^+ cells in CD8 T cells were decreased after PMN-MDSC transfer, with lowest level in tumor PMN-MDSC transferred group. However, in tumor tissues the IFN-γ^+ cells in CD8 T cells were not decreased after transferring DXM/LF PMN-MDSCs or Con PMN-MDSCs. Only tumor PMN-MDSC transfer reduced the IFN-γ^+ cells in CD8 T cells in tumor tissues. We revised our paper as follows:

Results

DXM/LF PMN-MDSCs presented improved survival capability, less tumor homing tendency and negative tumor promotive effects compared to tumor PMN-MDSCs

To analyze the effect of DXM/LF-induced PMN-MDSCs on both systemic and local antitumor immune responses, IFN-γ^+ CD8 T cells in PBMC and tumor tissue were tested by flow cytometers 24 hour after transfer of DXM/LF PMN-MDSCs, Con PMN-MDSCs, or tumor PMN-MDSCs into B16 tumor bearing mice, when the tumors were between 0.9 – 1.1 cm in diameter. PBS was used as vehicle control. In PBMC, compared with PBS group, the IFN-γ^+ cells in CD8 T cells were decreased after PMN-MDSC transfer, with lowest level in tumor PMN-MDSC transferred group. However, in tumor tissues the IFN-γ^+ cells in CD8 T cells were not decreased after

transferring DXM/LF PMN-MDSCs or Con PMN-MDSCs. Only tumor PMN-MDSC transfer reduced the IFN-γ^+ cells in CD8 T cells in tumor tissues. (Fig. 4E, Fig. S1C and Fig. S10).

3. The panel G in Fig. 7 is missing.

Response: we are sorry for this mistake. We added it in this version.

REVIEWERS' COMMENTS:

Reviewer #1 (Remarks to the Author):

none

Reviewer #2 (Remarks to the Author):

The manuscript has been improved greatly by adding additional experiments. I have no further comments.
